# Facilitators and barriers of life-space mobility in older adults with ischemic stroke: a descriptive qualitative study based on the COM-B

**DOI:** 10.3389/fpubh.2026.1811429

**Published:** 2026-07-13

**Authors:** Wangmo Dechen, Bo Xu, Hong Zeng, Xiaotong Fan, Chen Xu, Yanhui Qiu, Xihong Wang, Yuyu Qiu

**Affiliations:** 1 Wuxi School of Medicine, Jiangnan University, Wuxi, China; 2 Department of Nursing, People’s Hospital of Aba Tibetan and Qiang Autonomous Prefecture, Maerkang, Sichuan, China

**Keywords:** barriers, COM-B model, facilitators, ischemic stroke, life-space mobility, older

## Abstract

**Introduction:**

Older adults with ischemic stroke are particularly vulnerable to restricted life-space mobility, which is linked to adverse health outcomes. This study aimed to explore facilitators and barriers of life-space mobility among older adults with ischemic stroke using the COM-B model and to inform the development of targeted interventions.

**Methods:**

A descriptive qualitative design was adopted. Semi-structured, face-to-face interviews were conducted from June to September 2025 at the Hospital of Jiangnan University. Purposive maximum variation sampling was used to recruit 28 participants, including older adults with ischemic stroke and their caregivers. The interview guide was informed by the COM-B model, and data were analyzed using thematic analysis and mapped onto COM-B domains. This study was reported in accordance with the COREQ Checklist.

**Result:**

Factors associated with life-space mobility in older adults with ischemic stroke were categorized using the COM-B model: (1) capability factors, reflecting the influence of physical and psychological capability, including symptom burden, lack of knowledge, positive self-perception, and impaired self-determination; (2) opportunity factors, reflecting the influence of social and environmental opportunities, including family support, overprotective caregiving, social withdrawal, inadequate assistive-device and mobility support and environmental barriers; and (3) motivation factors, reflecting the influence of reflective and automatic motivation, including lack of motivation, fear of falling, and Self-limiting habits.

**Conclusion:**

This study applied of the COM-B model to identified the complex and multidimensional factors that influence life-space mobility in older adults with ischemic stroke, while also revealing the influence of the Chinese sociocultural context on life-space mobility in this population. These findings provide a basis for the design of comprehensive, targeted interventions across the capability, opportunity, and motivation domains to improve life-space mobility in older adults with ischemic stroke.

## 1 Introduction

Stroke is a leading cause of death and long-term disability worldwide, and its incidence increases markedly with advancing age (1). Ischemic stroke accounts for approximately 80% of all stroke cases and is particularly prevalent among older adults (2, 3). Older patients with ischemic stroke have been reported to experience motor impairments, such as decreased balance control, gait abnormalities, and impaired coordination (4). These impairments may lead to limitations in activities of daily living, including self-care, household tasks, and mobility-related daily activities, thereby reducing patients’ independence in everyday life (5, 6). Beyond activity limitations, stroke patients may also experience participation restrictions, such as reduced social interactions and limited involvement in family, occupational, and community activities, which further narrow their opportunities for outdoor mobility and community engagement (7, 8). After discharge, many patients also face multiple barriers, including insufficient support resources and limited accessibility and utilization of public facilities, which collectively reduce opportunities for community participation (9, 10). Notably, within the Chinese cultural context, family caregivers may adopt overprotective practices, such as taking over daily tasks or directly discouraging patients from going out, which can inadvertently reduce opportunities for independent activity and environmental adaptation (11). Meanwhile, stigma and perceived social discrimination after stroke may diminish willingness to engage socially and reduce interaction frequency, further confining activity to the home or nearby area (12). Fear of falling has also been common and may trigger self-restriction and avoidance of going out, leading to further contraction of activity space (13). Overall, mobility in older adults with ischemic stroke is shaped by multidimensional factors related to capability, environmental opportunities, and motivation, and is often characterized by substantially reduced life-space mobility in daily life.

Life-space mobility (LSM) refers to a multidimensional and dynamic indicator that captures how frequently, how far, and how independently an individual moves across different environmental settings in daily life (14). It reflects the complex interaction between personal functional capacity and social participation (15, 16). Such restriction in life-space mobility is closely associated with an increased risk of adverse health outcomes, including frailty, ADL disability, cognitive decline, depression, falls and fractures, and even mortality (17–21). Currently, studies have explored the factors associated with life-space mobility in older stroke patients. For instance, a cross-sectional study (22) conducted in Japan among community-dwelling individuals with stroke, with a mean age of 72.7 (7.4) years, found that 89.1% of participants had restricted life-space mobility and indicated that reduced activities of daily living, slower walking speed, and greater fear of falling were significant contributors to limited life-space mobility, suggesting that both physical and psychological factors play crucial roles in restricting life-space among older ischemic stroke patients. Additionally, a prospective observational study (23) including 59 community-dwelling patients with ischemic stroke, with a mean age of 70.8(9.7) years, revealed that stroke severity, falls efficacy, and comorbidity burden significantly affected the trajectories of life-space mobility recovery. However, existing research is predominantly quantitative and provides a limited understanding of patients’ experiences of life-space mobility and the barriers and facilitators. This gap is particularly salient in the Chinese context, where caregiving is predominantly family-centered amid substantial variations in community resources and environmental contexts, ultimately shaping distinct patterns of life-space mobility. Therefore, examining the barriers and facilitators of life-space mobility among older adults with ischemic stroke in China is essential for informing the development of targeted and effective interventions.

Theoretical frameworks are essential for understanding behavior and for guiding qualitative research in the development of complex interventions (24–26). The COM-B model (27) serves as an appropriate theoretical framework for comprehending the underlying reasons for behavior and their occurrence. The COM-B model comprises three core components: capability, opportunity, and motivation. Capability includes physical capability, which refers to functional skills and bodily strength, and psychological capability, which involves knowledge, cognitive processing, and the mental functions required to perform a behavior. Opportunity includes social opportunity shaped by interpersonal influences, cultural context, and available social support, as well as physical opportunity related to resources and environmental accessibility. Motivation covers both reflective motivations, involving conscious deliberation, planning, and evaluative beliefs, and automatic motivation, characterized by habitual responses, impulses, and emotional states. By integrating these components, the COM-B model enables a comprehensive analysis of the barriers that hinder behavior and the enablers that facilitate it. The COM-B model has been widely applied in research on chronic disease management and post-stroke health behaviors, identifying both facilitators and barriers to behavioral performance (28, 29). However, the application of the COM-B framework to examine barriers and facilitators of life-space mobility among older adults with ischemic stroke remains largely unexplored. Therefore, this study employed the COM-B framework to guide the qualitative, enabling a deeper understanding of the behavioral determinants.

This study aimed to examine the barriers and facilitators of life-space mobility from the perspectives of patients and caregivers, offering an in-depth understanding of the multidimensional factors shaping their experiences and informing the development of tailored interventions.

## 2 Methods

### 2.1 Design

A descriptive qualitative study design was adopted to explore the factors influencing life-space mobility among older adults with ischemic stroke (30). Guided by the naturalistic paradigm, this methodology enabled an in-depth understanding of participants’ experiences through face-to-face semi-structured interviews (31). The COM-B model was used to guide the development of the interview guide as well as data analysis (27). The study was reported in accordance with the Consolidated Criteria for Reporting Qualitative Research (COREQ) checklist (32), as detailed in Supplementary material 1. Ethical approval was obtained from the Medical Research Ethics Committee of Jiangnan University (Approval No. JNU202506RB046).

### 2.2 Setting

This study was conducted from June to September 2025 at the Jiangnan University Affiliated Hospital.

### 2.3 Participants

Purposive sampling, along with maximum variation sampling, was used to recruit older adults with ischemic stroke and their caregivers. To ensure diversity in the sample, recruitment efforts were made to include individuals with varying characteristics, including gender, age, duration since stroke, marital status, employment status, and current residence. The inclusion criteria for stroke patients were as follows: (1) a confirmed diagnosis of ischemic stroke based on established clinical criteria and cranial CT/MRI findings; (2) discharge to the community for at least 1 month; (3) age ≥60 years; (4) and could walk at least 16 m without physical assistance (33). Ischemic stroke patient exclusion criteria: (1) severe cognitive impairment or mental illness; (2) coexisting severe cardiac, pulmonary, or renal diseases; (3) communication barriers that hinder effective interaction. Caregiver inclusion criteria: (1) age ≥18 years; (2) currently providing care for a stroke survivor; (3) ability to communicate effectively. Caregiver exclusion criteria include paid caregivers and individuals with severe cognitive impairment or mental illness. All participants were volunteers and provided written informed consent before data collection.

The sample size was established by applying the principle of data saturation, with interviews continuing until no new categories or subcategories emerged. To assess whether saturation had been reached, the research team closely examined the evolving analyses, developing themes, and the depth and appropriateness of participant quotations. After the 28rd interview, data saturation was considered to have been reached, as the three subsequent interviews demonstrated no further emergence of new themes.

### 2.4 Data collection

This study utilized semi-structured, face-to-face in-depth interviews for data collection, with older persons with ischemic stroke and their family caregivers interviewed separately to allow them to freely share their perspectives on patients’ Life-space mobility. The interview guide was developed based on the COM-B model and thorough relevant literature reviews and pilot interviews. The guide was designed to capture psychological and physical capabilities, social and physical opportunities, and reflective as well as automatic motivations related to LSM, thereby ensuring a comprehensive understanding of its barriers and facilitators. The interview guide was iteratively developed through team discussions and pilot interviews. Two rounds of group discussion were conducted to identify potential determinants of life-space mobility in older adults with ischemic stroke. In addition, two stroke specialist nurses and one stroke clinician were consulted to further revise and refine the interview guide. Subsequently, we conducted pilot interviews with two patients and one caregiver, and the team discussed the findings and revised the interview guide accordingly. The feedback was used to refine and finalize the interview guide. Detailed components of the interview guide are provided in the Supplementary material 2.

The interviews were conducted in a quiet examination room in the neurology outpatient department after patients had completed their outpatient consultation, ensuring privacy and minimal disruption. The team members mainly consisted of an associate professor with experience in stroke research and graduate students majoring in gerontological nursing. The interviews were conducted by two female graduate students on the team, both trained in qualitative research and semi-structured interviewing techniques. In addition, the researchers conducted the interviews with no prior relationship to the participants. Ahead of commencing each interview, participants were informed of the study’s objective and that it would be audio-recorded. During the interviews, the researcher utilized clarification and repetition procedures as necessary to ensure comprehensive participant responses, while simultaneously observing and recording nonverbal signals like facial expressions, gestures, and pauses. The average duration of each interview is around 30 min. Following each interview, participants completed a demographic survey, and the researcher simultaneously reviewed their medical records to obtain Supplementary material 3 and recorded reflections in a journal.

### 2.5 Data analysis

Qualitative data were analyzed using Braun and Clarke’s thematic analysis (34). In the data preparation phase, the interviewer transcribed the interviews within 24 h, and the team member checked the transcripts against the recordings for accuracy. To ensure confidentiality, all collected data were de-identified, and participants were assigned unique pseudonyms. The de-identified data were imported into NVivo for systematic coding and thematic analysis. The analysis proceeded in two stages. Firstly, researchers familiarized themselves with the data through repeated reading, identified meaningful segments relevant to the research questions, and assigned codes. Similar codes were grouped to form potential themes, which were then reviewed against the complete dataset to ensure their coherence and validity. Secondly, two coders reviewed the themes and illustrative quotes and mapped them onto the COM-B framework. Any disagreements in coding were resolved through discussion with other team members to ensure reliability and consistency of the findings. To ensure methodological rigor, this study systematically employed multiple strategies, including member checking, comprehensive audit trails of the interview and coding processes, reflexive journaling, and detailed descriptions of the research context and participant characteristics, to enhance credibility, dependability, confirmability, and transferability (35).

## 3 Results

A total of 28 participants in this study, including older adults with ischemic stroke and their family caregivers. The patients were aged between 60 and 80 years, most were retired and resided in either urban or rural areas. Educational attainment among the patients was generally low to moderate, with primary and junior secondary education being the most common. The time since stroke onset was predominantly within the past year or between one and 5 years. Caregivers were predominantly adult children or spouses, most of whom were married and employed. Overall, the participant sample demonstrated considerable diversity in terms of age, residential setting, educational background, and caregiving roles. The characteristics of the participants are shown in Table 1, and the detailed participant information shown in the Appendix.

**Table 1:** Basic demographic characteristics of participants (*N* = 28).

Characteristics	Participants	
	Patients(*n* = 23)	Caregivers(*n* = 5)
Age (years)		
Mean	72.6	48.8
Range	60–88	38–75
Sex, n (%)		
Female	8(34.8)	3(60.0)
Male	15(65.2)	2(40.0)
Marital status, n (%)		
Married	19(86.6)	4(80.0)
Widowed	3(13.0)	1(20.0)
Remarried	1(4.3)	
Educational level, n (%)		
Primary and below	10(43.5)	1(20.0)
Middle school	9(39.1)	3(60.0)
Vocational secondary school	2(8.7)	0(0.0)
University and above	2(8.7)	1(20.0)
Current residence, n(%)		
rural	3(13.1)	2(40.0)
urban	20(87.0)	3(60.0)
Employment status, n(%)		
Employed	2(8.7)	3(60.0)
Not employed	3(13.0)	2(40.0)
Retired	18(78.3)	0(0.0)
Duration after stroke(years), n (%)		
<1	12(52.2)	NA
1–5	9(39.1)	
>5	2(8.7)	
Relationship with patients, n (%)		
Spouse	NA	1(20.0)
Parent		0(0.0)
Children		4(80.0)

**Figure 1:**
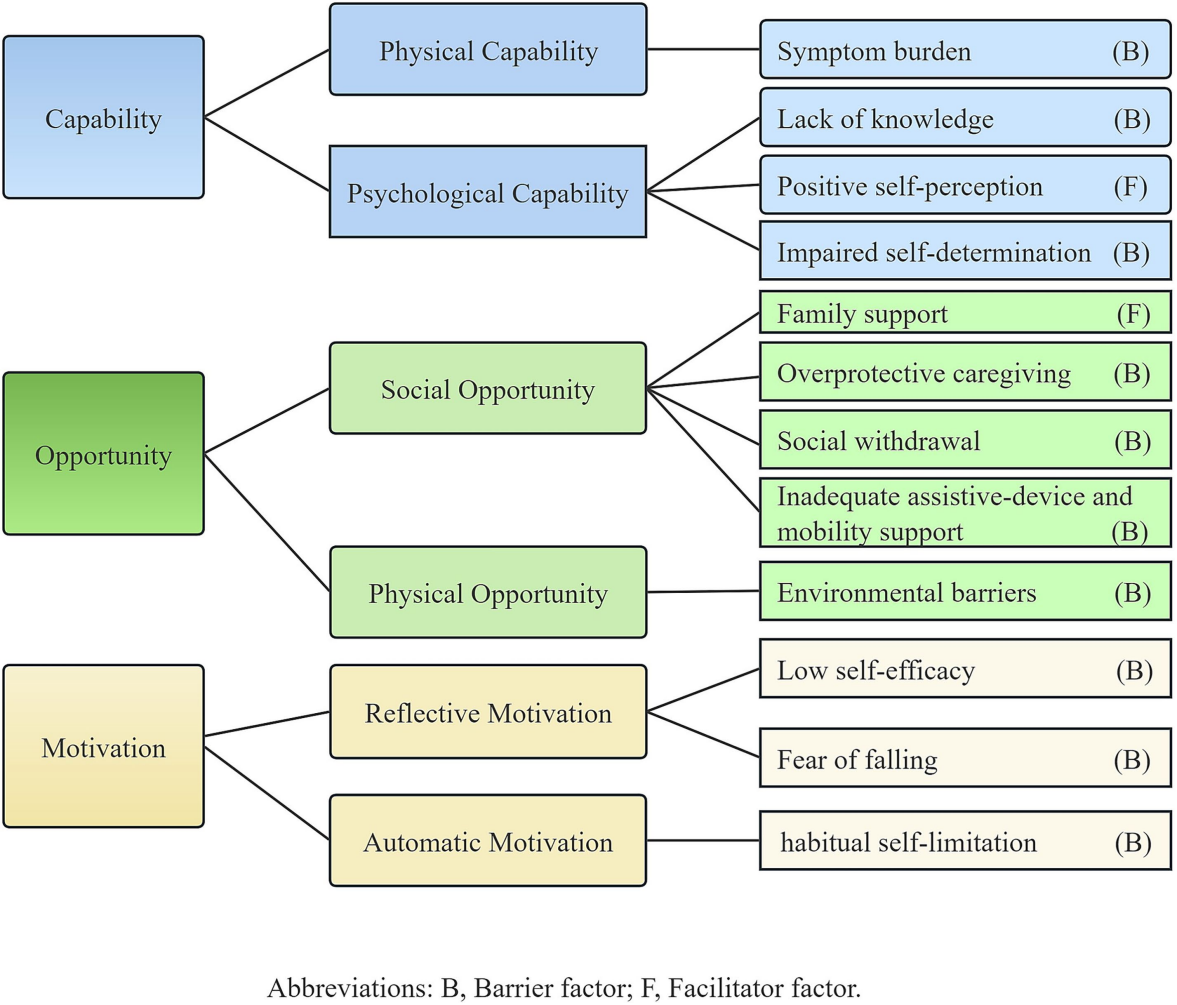
Factors influencing life-space mobility among older adults with ischemic stroke.

The study identified 12 subthemes across three primary categories aligned with the COM-B model, elucidating the factors influencing life-space mobility among older adults with ischemic stroke (Figure 1). The capability domain included symptom burden, lack of knowledge, positive self-perception, and impaired self-determination. Under the opportunity dimension, factors included family support, overprotective caregiving, social withdrawal, Inadequate assistive-device and mobility support and environmental barriers. Low self-efficacy, fear of falling, and self-limiting habits were observed under the motivation dimension.

### 3.1 Capability

#### 3.1.1 Subtheme: symptom burden

Most participants reported post-stroke limb weakness, reduced physical endurance, fatigue, pain, and dizziness or balance impairments, which resulted in marked difficulties in walking and performing daily activities. Some participants required the use of a cane for mobility, while others indicated that the overall symptom burden after stroke led them to avoid going long distances. These physical functional limitations not only constituted physiological barriers but also contributed to a pronounced pattern of long-distance avoidance behavior among participants.

*After walking for a while, my legs begin to ache and feel heavy, and I no longer have the strength to keep going. Therefore, I do not like going to places that are far away (P1).*

*When I walk farther or for a prolonged period, I start to feel dizzy. Although I can still manage with a cane, I do not go out very often (P15).*

*I have little strength in my upper limbs, so unless there is something necessary, I do not go out. At times, I cannot even brush my teeth independently and feel that I cannot clean properly. I also lack the strength to wash my face and experience lower back pain, so when I move around at home, I tend to want to sit down (P19).*

#### 3.1.2 Subtheme: lack of knowledge

Most participants demonstrated limited understanding or misconceptions regarding life-space mobility. Some were unable to articulate its meaning clearly and described it simply as being alive or life. Others reported limited capacity to acquire relevant knowledge and therefore lacked awareness of the concept of life-space mobility. A small number of participants further perceived life-space mobility after stroke as meaningless, believing that recovery depended solely on pharmacological treatment. Furthermore, limited smartphone proficiency restricted patients’ access to information channels, thereby hindering their acquisition of knowledge regarding life-space mobility.

*I am unfamiliar with the term’ life-space mobility’. Perhaps it refers to the act of living or one’s life. (P8).*

*I question the utility of increasing social participation or expanding one’s range of activities. If I feel unwell, I seek hospital care. Learning these concepts seems impractical. (P10).*

*I had no prior knowledge of life-space mobility because it was never explained to me, and I did not seek out such information via mobile devices. (P20).*

#### 3.1.3 Subtheme: positive self-perception

Positive perceptions of physical condition significantly influenced participants’ capability appraisals and recovery expectations. Participants who perceived gradual improvements in their functional abilities typically exhibited increased levels of activity and gained confidence from experiencing that their bodies remained capable of movement, which in turn made them more willing to maintain outdoor mobility and engagement in activities. Even when occasional mild lower-limb numbness occurred, they tended to interpret these symptoms as manageable and not restrictive to mobility.

*My physical condition has been much better recently compared with a while ago. There was a period when I was less active, but now I feel much better and can go out and walk around more. Getting outside also makes me feel more energetic. (P11).*

*I have been staying active during this period and going out regularly. Although my legs occasionally feel a bit numb, it does not affect my ability to be active. When my legs feel uncomfortable, I simply sit and rest for about half an hour, but it does not limit my activities. (P13).*

#### 3.1.4 Subtheme: impaired self-determination

Regarding decisions about life-space mobility, participants exhibited a marked lack of agency, frequently deferring decisions about the timing and destination of outdoor activities to family members. This decision-making impairment stemmed from a long-standing psychological dependency and the internal negation of one’s own planning capabilities. Moreover, patients’ daily activities were largely contingent on their relatives’ schedules, leading to a diminished ability to initiate and organize personal life activities independently. Such reliance on others’ planning gradually eroded patients’ capacity to proactively organize and manage their own daily lives.

*Where I go regularly is usually decided by my family members. (P6).*

*I’m not very good at arranging these things, so I follow my spouse’s decisions. (P14).*

*How long I go out is usually determined by my family’s schedule, most often in the morning or after dinner. (P20).*

### 3.2 Opportunity

#### 3.2.1 Subtheme: family support

Most participants identified familial support and emotional companionship as pivotal factors in promoting post-stroke mobility recovery and life-space mobility. Specifically, instrumental assistance provided by family members, such as accompanying the patient during walks and prompting exercise, facilitated the gradual restoration of functional capacity. Furthermore, emotional support enhanced patients’ confidence and willingness to engage in outdoor activities and social interactions.

*My wife frequently reminds me to exercise and accompanies me on outings. This has been instrumental to my recovery. (P13).*

*My son and daughter-in-law take me to family gatherings or join me for meals at home. Their companionship has significantly improved my mood. (P12).*

#### 3.2.2 Subtheme: overprotective caregiving

A significant number of patients resided in a home environment characterized by hyper-vigilant or overprotective caregiving. Specifically, family members frequently implemented constant supervision or rotational caregiving shifts to prevent potential accidents during outdoor excursions. Consequently, independent mobility was largely restricted. In the absence of a companion, patients were typically expected to remain indoors. Furthermore, caregivers assumed responsibility for the majority of domestic chores and personal care tasks. This high level of protective care inadvertently constrained the patients’ autonomous activities and limited their opportunities for functional engagement.

*We never let him go out alone. Someone has to accompany him for us to feel at ease. When everyone is busy, he stays at home. There is really nothing he needs to do outside, and staying at home without falling is the best thing he can do (C2).*

*My spouse handles almost all the housework and assists me with personal hygiene, such as washing my face and feet, as well as doing the laundry. (P10).*

#### 3.2.3 Subtheme: social withdrawal

Following a stroke, the majority of patients experienced a marked contraction of their social networks. For some, diminished muscle strength or impaired motor coordination rendered pre-stroke leisure activities, such as card playing or dancing, increasingly difficult to maintain. Others proactively curtailed social gatherings, such as alcohol consumption. Furthermore, concerns regarding negative social evaluation or feelings of embarrassment in group settings emerged as significant barriers. Additionally, a minority of respondents reported a preference for reduced activities outside the home to avoid imposing a perceived burden on others.

*My hands and feet are no longer agile, so playing cards has become inconvenient, and going out to play together no longer feels meaningful. (P16).*

*Before I became ill, I often went out with close friends to socialize and have a few drinks. Now my body cannot handle it, and since I can no longer drink alcohol, I do not go anymore. (P21).*

*You see, my left leg does not move well anymore, and I cannot dance in the square like I used to. Others would laugh at me, and it feels embarrassing. (P5).*

#### 3.2.4 Subtheme: inadequate assistive-device and mobility support

Some participants reported a lack of systematic and professional guidance regarding the use of assistive mobility devices following their stroke. This deficit was particularly evident when navigating complex environments, such as stairs and ramps, forcing patients to rely on trial-and-error to develop self-taught compensatory strategies. Such a lack of instruction not only heightened perceived insecurity during excursions but also attenuated their proactivity to go outdoors. Furthermore, transportation accessibility emerged as a significant barrier. Patients identified the loss of driving ability and the challenges of public transit as key barriers, forcing a significant dependency on family support for their transportation needs. Consequently, these factors further constricted the expansion of their life-space mobility.

*No one instructed me on how to use the mobility aid, especially when navigating stairs, I had to figure it out gradually through trial and error. (P18).*

*Transportation also poses a significant challenge for me. Since my illness, driving has become impossible, and using public transit involves numerous transfers. Consequently, I am heavily dependent on my family for any outings. (P17).*

#### 3.2.5 Subtheme: environmental barriers

Structural obstacles within the residential environment significantly restrict patients’ life-space mobility. Patients reported challenges such as difficulty navigating stairs, inadequate lighting, and physical obstructions, which directly undermined their safety and reduced their motivation to leave home. Additionally, ongoing neighborhood renovations were also cited by a few participants as an environmental barrier to life-space mobility.

*I live on the third floor and have to hold onto the handrail when going downstairs. The corridor lights are voice-activated, and at night I worry about missing a step. (P4).*

*We live in resettlement housing, and recently the neighborhood has been undergoing renovation. Stones have been piled up on the ramp at the entrance, making it difficult to use assistive devices, so I have rarely gone out over the past month. (P22).*

### 3.3 Motivation

#### 3.3.1 Subtheme: low self-efficacy

Some patients demonstrated markedly reduced self-efficacy, reflected in limited confidence in their functional abilities, reduced persistence when encountering challenges, and a tendency to discontinue efforts prematurely. This phenomenon was particularly pronounced among older adults respondents, who frequently maintained a pessimistic outlook regarding rehabilitation outcomes and tended to withdraw from activities when encountering difficulties. Furthermore, some patients displayed an indifference toward life goals and a lack of volition to improve their current status, manifesting a passive or defeatist attitude.

*I do not really expect much improvement anyway. (P7).*

*He does not really have the mindset to push through difficulties. After just a few steps, he feels tired. We’ve encouraged him many times and accompanied him repeatedly, but nothing seems to work. He just stays there and does not like to communicate with us either. (C1).*

*If I’m no longer able to walk at all, then maybe I’ll just go to a nursing home. There’s nothing in particular that I really want to do anyway. (P8).*

*At my age, I’ll just do whatever I’m still able to do. I do not want to push myself anymore. (P15).*

#### 3.3.2 Subtheme: fear of falling

Participants commonly reported that a persistent fear of falling significantly restricted their activity range. The apprehension of losing balance or falling during ambulation led to a reduced frequency of outdoor activity. Some participants completely avoided leaving their homes for extended periods, confining their movement to short distances within the indoor environment. Collectively, this fear of falling resulted in a marked decline in daily activity levels and a subsequent contraction in life-space mobility.

*I’ve barely gone outside lately, simply because I was afraid of falling. (P9).*

*I am always extremely cautious when walking because my greatest fear is falling. Now, I only dare to walk back and forth inside the house and rarely venture outdoors. (P17).*

#### 3.3.3 Subtheme: self-limiting habits

The lifestyle habits of most patients significantly impede their life-space mobility. This was reflected in a preference for remaining in familiar environments, a reluctance to adopt new assistive devices, and a tendency to avoid noisy or crowded public spaces. Collectively, these habitual patterns served as barriers, significantly restricting the expansion of patients’ life-space mobility.

*I am habituated to sitting on the balcony and looking out the window, or at most, sitting just outside the building. I do not usually go anywhere else. (P19).*

*I am not accustomed to using those devices. Using a walker or a cane is not something I’m used to, and it makes my condition look far more severe than it is. (P22).*

*He has developed a fixed habit of sitting on the sofa all day, keeping the remote and water glass within immediate reach. (C3).*

*I tend to avoid community activities as I find them too noisy and prefer a quieter setting. (P8).*

## 4 Discussion

To our knowledge, this qualitative study is the first to apply the COM-B model to explore, from the viewpoints of older adults with ischemic stroke and their caregivers, the factors that obstruct or facilitate life-space mobility. Guided by the COM-B model, patients’ life-space mobility was examined across the dimensions of capability, opportunity, and motivation. Notably, the findings also show how China-specific sociocultural factors impact life-space mobility, providing valuable information for the creation of interventions that are both targeted and culturally appropriate.

### 4.1 Capability

This study suggested that physical capacity was an important contributor to life-space mobility among older adults with ischemic stroke. Consistent with previous research, impairments such as lower-limb weakness, balance dysfunction, and reduced gait speed have been significantly associated with restricted life-space mobility in this population (22, 23). In addition, patients’ accounts indicated that these physical limitations were often accompanied by activity-avoidance tendencies. Activities such as walking longer distances or climbing stairs were often perceived as physically exhausting, leading patients to reduce their engagement in these activities. In such situations, caregivers may help facilitate appropriate rest during activities outside the home, such as by using a wheelchair to provide intermittent rest when public seating is unavailable and the surrounding infrastructure allows wheelchair use. Accordingly, interventions that strengthen physical capacity may be particularly relevant. Enhancing lower-limb strength, improving gait speed, and providing balance training may increase functional capacity, while pacing strategies may help mitigate fatigue and reduce avoidance driven by symptom burden (36, 37). Together, these approaches may improve activity tolerance and perceived safety, thereby reducing avoidance behaviors and supporting broader life-space mobility.

In addition to physical capacity, life-space mobility in older adults with ischemic stroke was strongly influenced by psychological capability. This was reflected in both barriers and facilitators: a lack of knowledge related to life-space mobility and impaired self-determination emerged as key barriers, whereas positive self-perception functioned as a facilitating factor. Specifically, some participants demonstrated limited understanding or misconceptions regarding the concept of life-space mobility and failed to recognize its relevance to health maintenance and functional recovery. A lack of knowledge may undermine patients’ judgment, thereby adversely influencing their behavior (38). Accordingly, improving patients’ knowledge of life-space mobility should be regarded as an important prerequisite for enhancing life-space mobility. Previous studies have shown that targeted educational interventions can effectively improve patients’ knowledge and promote the adoption of health-related behaviors (39). Building on this evidence, health education focused on life-space mobility may help patients better understand the significance and feasibility of different levels of life-space mobility. Moreover, structured knowledge-based interventions may gradually correct misconceptions, thereby strengthening patients’ intention to act (40). Beyond patient education, caregiver education should also be considered, as caregivers play an important role in supporting patients’ activities of daily living after stroke (41). Previous studies have shown that caregiver-focused education may improve patients’ functional recovery and activities of daily living (42–44).

This study identified positive self-perception as an important psychological capability facilitating life-space mobility among older adults with ischemic stroke. Participants who subjectively perceived improvements in their physical functioning generally expressed greater willingness to engage in activity and, despite mild residual symptoms, were more likely to expand their activity range and participate in social activities. This finding aligned with previous evidence indicating that positive self-perceptions could enhance motivation to initiate and sustain activity participation (45). Furthermore, existing research has indicated that psychological factors are closely related to the extent of life-space mobility. Relevant stroke studies have also suggested that psychological support may help promote rehabilitation engagement and improve functional outcomes (46). Moreover, psychosocial interventions have been implemented in stroke populations and have shown promising effects (47, 48). Therefore, interventions targeting life-space mobility should consider strengthening positive self-perception and related psychological factors, as these may enhance patients’ willingness to engage in activities and support the gradual expansion of range.

Beyond the above, patients’ accounts also highlighted the role of self-determination in shaping life-space mobility. Most patients exhibited impaired self-determination, which was associated with lower levels of life-space mobility. Some patients rarely made independent decisions about whether to go out or where to go. Instead, such decisions were often made by family members or could only be implemented with family accompaniment. This phenomenon may be partly explained by the Chinese cultural context, in which family members commonly assume protective or guiding roles for older relatives. While well-intentioned, such practices may inadvertently undermine older patients’ autonomy in everyday decision-making, erode their sense of agency and hinder their engagement in post-stroke life (49, 50). Accordingly, enhancing patients’ capacity for self-decision-making may be warranted. First, behavioral training could be implemented to strengthen decision-making skills related to out-of-home activities (51). Specifically, behavioral training may include caregiver training provided by specialized rehabilitation therapists to ensure safe assistance with walking and transfers, thereby enhancing caregivers’ confidence (52). Meanwhile, family members should be guided to cultivate patients’ autonomy and self-decision making in daily life activities, as enhancing stroke survivors’ autonomy and self-advocacy has been associated with greater engagement in social and outdoor activities (53). Finally, by progressively returning decisions related to life-space mobility to patients, autonomy in daily life may be strengthened, ultimately supporting the expansion of life-space mobility.

### 4.2 Opportunity

The study found the complex influence of opportunity on life-space mobility among older adults with ischemic stroke, encompassing family support, overprotective caregiving, social withdrawal, Inadequate assistive-device and mobility support and environmental barriers. Among these factors, positive family support is a facilitator of post-stroke functional recovery and engagement in the community. Life-space mobility not only reflects patients’ actual range of movement but is also closely associated with social participation and community integration (54). Previous studies have shown that family support can significantly enhance community integration after stroke by increasing patients’ confidence in outdoor activities, facilitating access to social resources, and providing practical assistance in daily mobility and participation (55). To more effectively harness the positive role of family support, family-centered supportive participation programs could be developed (56). Through guided family assistance that helps patients gradually expand their mobility range, these may foster a progressive return to social participation, ultimately leading to enhanced life-space mobility.

Our study identified overprotective caregiving as a barrier to life-space mobility among older adults with ischemic stroke. The majority of patients’ families adopted a highly protective caregiving pattern, in which patients were discouraged from going outdoors independently and caregivers routinely performed daily tasks on their behalf. The emergence of this caregiving model may be closely tied to the deeply rooted ethics of familial responsibility and the cultural tendency toward risk avoidance in traditional Chinese society. Specifically, under the influence of Confucian filial piety, meticulous caregiving is often regarded as a moral obligation and virtue, while any potential safety risks encountered by the patient are interpreted as signs of familial negligence (57). Consequently, caregivers tend to focus exclusively on ensuring safety while neglecting the patient’s autonomy activities. Ultimately, such prolonged dependence may contribute to a further decline in life-space mobility.

This study also identified social withdrawal as a barrier factor to life-space mobility in China. Some participants reduced social participation due to physical impairments, whereas others avoided social activities out of fear of negative evaluation and stigma. Certain participants reported deliberately limiting social contact to avoid becoming a burden to others. This study further found that social withdrawal was mainly driven by stigma and perceived burden. Stigma stems from the rupture of social identity and the visible changes in physical function following stroke, leading patients to feel stigmatized and anticipate potential social discrimination (58). This fear of being labeled prompts them to avoid social activities where their disability may be exposed, thereby contributing to social withdrawal (59). Perceived burden may be rooted in the collectivist cultural values of Chinese society, which promote attending to others’ needs as virtuous while regarding self-focused behavior as selfish (60). Under such cultural norms, patients tended to minimize reliance on others by withdrawing from social interactions, thereby exhibiting pronounced social withdrawal. Such social withdrawal behaviors not only impose direct constraints on life-space mobility but also hinder patients from accessing valuable support, feedback, and experiential learning through interpersonal interactions. Notably, peer support may also be considered as a potential strategy to address this barrier. Through shared experiences, emotional encouragement, coping support, and mutual learning, peer-based support may help patients reduce feelings of isolation and rebuild confidence in social participation (61, 62).

In addition, this study found that inadequate assistive-device and mobility support and environmental barriers further constrain life-space mobility among older adults with ischemic stroke. Following hospital discharge, many patients were unable to drive and encountered substantial challenges in accessing public transportation, which significantly restricted their independent life-space mobility. Compounding this issue, the lack of professional instruction and scenario-specific training in the use of assistive devices further diminished their capacity to move within their living environment. At the same time, community renovation processes often involve the temporary occupation of accessible pathways, creating environmental obstacles to outdoor participation. Consistent with prior research (63), a disconnect persists between institutional healthcare services and community-based support during the transition from hospital to daily life. In particular, this gap is frequently exacerbated by deficiencies in community planning and transport infrastructure. Addressing these gaps requires the development of an integrated support network linking hospitals, communities, and families, allocating resources to strengthen transitional care, and systematically incorporating stroke-friendly principles into community infrastructure and transportation planning to enhance life-space mobility in this population.

### 4.3 Motivation

Our study also found that motivation played a crucial role in shaping patients’ life-space mobility, particularly through low self-efficacy, fear of falling, and habitual self-limitation. Among these factors, low self-efficacy emerged as a barrier to life-space mobility. Some patients, lacking confidence in functional recovery, exhibited a passive coping attitude that diminished self-efficacy and directly suppressed their willingness to expand life-space mobility, resulting in persistently restricted activity ranges. This finding was consistent with evidence from chronic disease rehabilitation research, which demonstrated that self-efficacy was a significant determinant of health-related behaviors (64). Patients’ self-efficacy shapes how they respond to challenges related to life-space mobility. Therefore, greater emphasis should be placed on self-efficacy supportive strategies, incorporating approaches such as stepwise goal setting and positive behavioral feedback to help patients gradually rebuild confidence in their own capabilities and strengthen their intrinsic motivation to actively expand life-space mobility.

In addition, this study found that fear of falling is common among patients with ischemic stroke. Most participants reported fearing loss of balance or falling while walking, which led them to deliberately reduce outdoor activities or even remain confined to the home for extended periods. Fear of falling is commonly observed after stroke and has been consistently linked to restricted life-space mobility in older adults (65, 66). This study further found that, among older adults with ischemic stroke, avoidance behaviors driven by fear of falling may initiate a vicious cycle of life-space mobility contraction and reduce physical activity, ultimately hindering recovery. To address this issue, graded outdoor activity plans combined with virtual scenario–based training may be implemented by rehabilitation professionals to progressively alleviate fear of falling, facilitate the expansion of life-space mobility, and ultimately improve overall rehabilitation outcomes and the quality of life.

In addition to the factors noted above, we found that self-limiting habits also influenced patients’ life-space mobility. Some participants were habituated to confining their activities to familiar settings, such as balconies or sofas, and showed little motivation to go outdoors. Some participants described a habitual reliance on unaided mobility and showed resistance to using new assistive devices. Consistent with previous research, stroke survivors may experience resistance to, or difficulties accepting, the use of assistive devices after stroke (67, 68). In addition, the onset of stroke may disrupt patients’ established lifestyle patterns, making it difficult for them to adapt to new circumstances and leading them to rely more on familiar environments and habitual behaviors (69). Although such entrenched habits may provide comfort, they could also lead to a gradual narrowing of mobility range and reduced functional adaptability, ultimately reinforcing long-term restrictions in life-space mobility. Therefore, interventions should be anchored within patients’ existing comfort zones and guide them to systematically expand activity boundaries in familiar environments, thereby facilitating the gradual formation of positive new behavioral patterns and progressively enhancing life-space mobility.

## 5 Strengths and limitations

This study has several strengths. First, it applied the COM-B model to systematically explore determinants of life-space mobility among older adults with ischemic stroke in terms of physical and psychological capability, social and physical opportunity, and reflective and automatic motivation. This approach not only helped identify key barriers and facilitators but also provided a theory-informed basis for developing targeted intervention strategies. Second, incorporating both patients and family caregivers enabled a more comprehensive understanding of life-space mobility from both individual and family caregiving perspectives. However, this study also has several limitations. First, participants were recruited from a single hospital. Future research could recruit participants from multiple hospitals and community settings. Second, although the COM-B model provided a useful framework for organizing behavioral determinants, demographic characteristics, such as sex, marital status, residence, and employment status, were not fully explored as independent themes due to the qualitative design and limited sample size. Future studies with larger and more diverse samples are needed to further examine the potential influence of these demographic factors on life-space mobility.

## 6 Conclusion

This study explored the influencing factors affecting life-space mobility in older adults with ischemic stroke through qualitative interviews based on the COM-B framework. The results showed that, in addition to individual capability-related factors, opportunity-related factors and motivation-related factors also had a significant impact on patients’ life-space mobility. To improve life-space mobility, multiple behavioral determinants across the dimensions of capability, opportunity, and motivation must be recognized and addressed. Therefore, a multidimensional life-space mobility intervention program should be developed, consisting of strategies to enhance patients’ capability, expand supportive opportunities, and strengthen motivational resources. This study provides a reference for the development of comprehensive intervention strategies to improve life-space mobility in older adults with ischemic stroke. Moreover, to support the long-term sustainability of the intervention program, collaboration with healthcare systems and public-space management sectors may be considered to improve accessible transportation and inclusive, stroke-friendly community spaces. In addition, co-designing the intervention with key stakeholders, including patients, caregivers, physicians, and rehabilitation specialists, may help ensure the intervention’s feasibility and acceptability.

## Data Availability

The original contributions presented in the study are included in the article/Supplementary material, further inquiries can be directed to the corresponding authors.
