# Supplementary material for: Facilitators and barriers of life-space mobility in older adults with ischemic stroke: a descriptive qualitative study based on the COM-B
Source: Front Public Health. 2026 Jul 13;14:1811429. doi: 10.3389/fpubh.2026.1811429 (PMC13402188; doi:10.3389/fpubh.2026.1811429)
Supplement: Supplementary file 1 [file Supplementary_file_1.docx]

**COREQ (COnsolidated criteria for REporting Qualitative research) Checklist**

A checklist of items that should be included in reports of qualitative research. You must report the page number in your manuscript where you consider each of the items listed in this checklist. If you have not included this information, either revise your manuscript accordingly before submitting or note N/A.

| **Topic** | **Item No.** | **Guide Questions/Description** | **Reported on Page No/ section** |
| --- | --- | --- | --- |
| **Domain 1: Research team**  **and reﬁexivity** | | | |
| *Personal characteristics* | | | |
| Interviewer/facilitator | 1 | Which author/s conducted the interview or focus group? | P3/Data collection |
| Credentials | 2 | What were the researcher’s credentials? E.g. PhD, MD | P3/Data collection |
| Occupation | 3 | What was their occupation at the time of the study? | P3/Data collection |
| Gender | 4 | Was the researcher male or female? | P3/Data collection |
| Experience and training | 5 | What experience or training did the researcher have? | P3/Data collection |
| *Relationship with*  *participants* | | | |
| Relationship established | 6 | Was a relationship established prior to study commencement? | P3/Data collection |
| Participant knowledge of the interviewer | 7 | What did the participants know about the researcher? e.g. personal goals, reasons for doing the research | P3/Data collection |
| Interviewer characteristics | 8 | What characteristics were reported about the inter viewer/facilitator? e.g. Bias, assumptions, reasons and interests in the research topic | P3/Data collection |
| **Domain 2: Study design** | | | |
| *Theoretical framework* | | | |
| Methodological orientation and Theory | 9 | What methodological orientation was stated to underpin the study? e.g. grounded theory, discourse analysis, ethnography, phenomenology,  content analysis | P2/Design, P3/Data analysis |
| *Participant selection* | | | |
| Sampling | 10 | How were participants selected? e.g. purposive, convenience, consecutive, snowball | P3/Participants |
| Method of approach | 11 | How were participants approached? e.g. face-to-face, telephone, mail, email | P3/Data collection |
| Sample size | 12 | How many participants were in the study? | P3/Participants,P3/Results |
| Non-participation | 13 | How many people refused to participate or dropped out? Reasons? | N/A |
| *Setting* | | | |
| Setting of data collection | 14 | Where was the data collected? e.g. home, clinic, workplace | P3/Data collection |
| Presence of non- participants | 15 | Was anyone else present besides the participants and researchers? | P3/Data collection |
| Description of sample | 16 | What are the important characteristics of the sample? e.g. demographic data, date | P3/Results |
| *Data collection* | | | |
| Interview guide | 17 | Were questions, prompts, guides provided by the authors? Was it pilot tested? | P3/Data collection |
| Repeat interviews | 18 | Were repeat inter views carried out? If yes, how many? | N/A |
| Audio/visual recording | 19 | Did the research use audio or visual recording to collect the data? | P3/Data collection |
| Field notes | 20 | Were ﬁeld notes made during and/or after the inter view or focus group? | P3/Data collection |
| Duration | 21 | What was the duration of the inter views or focus group? | P3/Data collection |
| Data saturation | 22 | Was data saturation discussed? | P3/Participants |
| Transcripts returned | 23 | Were transcripts returned to participants for comment and/or | N/A |

| **Topic** | **Item No.** | **Guide Questions/Description** | **Reported on Page No.** |
| --- | --- | --- | --- |
|  |  | correction? |  |
| **Domain 3: analysis and**  **ﬁndings** | | | |
| *Data analysis* | | | |
| Number of data coders | 24 | How many data coders coded the data? | P3/Data analysis |
| Description of the coding tree | 25 | Did authors provide a description of the coding tree? | P3/Data analysis |
| Derivation of themes | 26 | Were themes identiﬁed in advance or derived from the data? | P3/Data analysis |
| Software | 27 | What software, if applicable, was used to manage the data? | P3/Data analysis |
| Participant checking | 28 | Did participants provide feedback on the ﬁndings? | P3/Data analysis |
| *Reporting* | | | |
| Quotations presented | 29 | Were participant quotations presented to illustrate the themes/ﬁndings? Was each quotation identiﬁed? e.g. participant number | P4-7/Results |
| Data and ﬁndings consistent | 30 | Was there consistency between the data presented and the ﬁndings? | P4-7/Results |
| Clarity of major themes | 31 | Were major themes clearly presented in the ﬁndings? | P4-7/Results |
| Clarity of minor themes | 32 | Is there a description of diverse cases or discussion of minor themes? | P4-7/Results |

Developed from: Tong A, Sainsbury P, Craig J. Consolidated criteria for reporting qualitative research (COREQ): a 32-item checklist for interviews and focus groups. *International Journal for Quality in Health Care*. 2007. Volume 19, Number 6: pp. 349 – 357

**Once you have completed this checklist, please save a copy and upload it as part of your submission. DO NOT include this checklist as part of the main manuscript document. It must be uploaded as a separate file.**
