# Supplementary material for: Facilitators and barriers of life-space mobility in older adults with ischemic stroke: a descriptive qualitative study based on the COM-B
Source: Front Public Health. 2026 Jul 13;14:1811429. doi: 10.3389/fpubh.2026.1811429 (PMC13402188; doi:10.3389/fpubh.2026.1811429)
Supplement: Supplementary file 2 [file Supplementary_file_2.docx]

Supplementary Material 2

# Detailed components of the interview guidere provided in Tables 1 and 2.

**Table 1.** Patient's interview guide

| **COM-B Model** | | **Patients Questions** |
| --- | --- | --- |
| **Capability** | Physical  Capability | 1. What physical changes have you experienced after the stroke that limit your ability to go outdoors? |
|  | Psychological Capability | 1. Could you describe your understanding of life-space mobility? What factors do you believe influence your life-space mobility? 2. How do you learn about ways to maintain or improve your ability to go outdoors and engage in mobility activities? |
| **Opportunity** | Social  Opportunity | 1. To what extent do you feel that your family members, friends, healthcare providers, or governmental/social support systems influence your daily mobility? 2. What roles do you think family members and healthcare professionals should play in supporting or managing your life-space mobility? 3. Do societal culture or traditional customs influence the way you move within your life-space or your mobility abilities? If so, could you explain how? |
|  | Physical Opportunity | 1. In your opinion, what aspects of your home, community, hospital, or broader social environment help or hinder your daily life-space mobility? |
| **Motivation** | Reflective Motivation | 1. What are your personal goals regarding improving your life-space mobility? 2. In what ways do you think life-space mobility brings positive or negative impacts to your daily life? 3. What attitudes do you hold toward your own ability to move within your life-space? Could you elaborate? |
|  | Automatic Motivation | 1. What internal factors, such as emotions, habits, or personality traits, affect your life-space mobility? |

**Table 2.** Caregiver's interview guide

| **COM-B Model** | | **Caregivers Questions** |
| --- | --- | --- |
| **Capability** | Physical  Capability | 1. What physical factors have you observed among the stroke patients you care for that may influence their ability to move within their life-space mobility? |
|  | Psychological Capability | 1. How well do you and the patients under your care understand the concept of life-space mobility? 2. To what extent do you and your patients understand how to maintain or improve their ability to go outdoors or engage in mobility-related activities? |
| **Opportunity** | Social  Opportunity | 1. From your observations, how do family members, friends, hospitals, or governmental and community services influence patients’ mobility in their daily life-space? 2. In managing and supporting patients’ life-space mobility, what roles should family members and healthcare professionals play? 3. Do cultural or traditional practices influence the way patients move within their life-space or affect their mobility capacity? Please explain why. 4. What environmental factors within the family, hospital, or broader community help facilitate or hinder stroke patients' mobility in their daily life-space? Could you provide some examples? Do societal culture or traditional customs influence the way you move within your life-space mobility? If so, could you explain how? |
|  | Physical Opportunity | 1. what aspects of your home, community, hospital, or broader social environment help or hinder patients daily life-space mobility? |
| **Motivation** | Reflective Motivation | 1. What positive or negative impacts do you think life-space mobility may have on patients’ physical and psychological recovery? Please share your perspective. 2. Based on your caregiving experience, what attitudes do patients typically hold toward their own mobility within their life-space? How do these attitudes influence their rehabilitation? |
|  | Automatic Motivation | 11.During your caregiving process, have you noticed any spontaneous personal factors, such as emotions, personality traits, or habitual behaviors, that affect patients’ daily mobility? Could you provide some examples? |
