# Supplementary material for: Facilitators and barriers of life-space mobility in older adults with ischemic stroke: a descriptive qualitative study based on the COM-B
Source: Front Public Health. 2026 Jul 13;14:1811429. doi: 10.3389/fpubh.2026.1811429 (PMC13402188; doi:10.3389/fpubh.2026.1811429)
Supplement: Supplementary file 3 [file Supplementary_file_3.docx]

Supplementary Material 3

## Table 1 Basic demographic characteristics of patients (N = 23)

| **No** | **Gender** | **Age（years）** | **Marital status** | **Household members** | **Duration after stroke（months）** | **Educational level** | **Employment status** | **Current residence** |
| --- | --- | --- | --- | --- | --- | --- | --- | --- |
| P1 | Male | 62 | Married | Spouse, Children | 6 | Middle School | Retired | Urban |
| P2 | Male | 76 | Married | Spouse, Children | 46 | Primary school | Retired | Rural |
| P3 | Female | 75 | Widowed | Children | 156 | Vocational school | Retired | Urban |
| P4 | Male | 70 | Married | Spouse | 21 | College diploma | Retired | Urban |
| P5 | Female | 61 | Married | Spouse | 2 | College diploma | Retired | Urban |
| P6 | Female | 76 | Widowed | Living alone | 6 | Middle School | Retired | Urban |
| P7 | Female | 64 | Married | Spouse | 11 | Middle School | Retired | Urban |
| P8 | Male | 79 | Married | Spouse | 18 | Primary school | Farmer | Rural |
| P9 | Male | 88 | Married | Spouse | 4 | None | No-job | Urban |
| P10 | Female | 60 | Married | Spouse | 6 | Middle School | Farmer | Urban |
| P11 | Male | 81 | Married | Spouse, Children | 9 | Middle School | Retired | Urban |
| P12 | Male | 70 | Married | Spouse | 3 | Primary school | Cleaner | Urban |
| P13 | Male | 73 | Married | Spouse | 28 | Primary school | Retired | Urban |
| P14 | Male | 76 | Married | Spouse | 63 | Middle School | Retired | Urban |
| P15 | Male | 67 | Remarried | Spouse | 5 | Primary school | Do-job | Urban |
| P16 | Female | 80 | Widowed | Children | 22 | Middle School | Retired | Urban |
| P17 | Male | 65 | Married | Spouse | 24 | Primary school | Retired | Rural |
| P18 | Male | 84 | Married | Spouse | 5 | Primary school | Retired | Urban |
| P19 | Male | 78 | Married | Children | 7 | Vocational school | Retired | Urban |
| P20 | Female | 79 | Married | Spouse | 48 | Middle School | Retired | Urban |
| P21 | Male | 65 | Married | Spouse | 23 | Primary school | Retired | Urban |
| P22 | Male | 77 | Married | Spouse | 8 | Primary school | Retired | Urban |
| P23 | Female | 65 | Married | Children | 11 | Middle School | Retired | Urban |

## Tables 2 Basic demographic characteristics of caregivers (N = 5)

| **No** | **Gender** | **Age（years）** | **Marital status** | **Educational level** | **Relationship with patients** | **Current residence** |
| --- | --- | --- | --- | --- | --- | --- |
| P1 | Male | 48 | Married | Middle School | Children | Urban |
| P2 | Female | 38 | Married | College diploma | Daughter-in-law | Rural |
| P3 | Female | 75 | Married | Primary school | Spouse | Urban |
| P4 | Male | 41 | Widowed | Middle School | Children | Urban |
| P5 | Female | 42 | Married | Middle School | Children | Urban |
